# Supplementary material for: Selection of a de novo gene that can promote survival of Escherichia coli by modulating protein homeostasis pathways
Source: Nat Ecol Evol. 2023 Nov 9;7(12):2067–79. doi: 10.1038/s41559-023-02224-4 (PMC10697842; doi:10.1038/s41559-023-02224-4)
Supplement: Supplementary file 1 — Reporting Summary [file 41559_2023_2224_MOESM1_ESM.pdf]

nature portfolio

Corresponding author(s): Laub, Michael

Last updated by author(s): Sep 6, 2023

Reporting Summary

Nature Portfolio wishes to improve the reproducibility of the work that we publish. This form provides structure for consistency and transparency in reporting. For further information on Nature Portfolio policies, see our Editorial Policies and the Editorial Policy Checklist.

Statistics

For all statistical analyses, confirm that the following items are present in the figure legend, table legend, main text, or Methods section.

n/a	Confirmed	
☐	☒	The exact sample size (*n*) for each experimental group/condition, given as a discrete number and unit of measurement
☐	☒	A statement on whether measurements were taken from distinct samples or whether the same sample was measured repeatedly
☐	☒	The statistical test(s) used AND whether they are one- or two-sided<br>*Only common tests should be described solely by name; describe more complex techniques in the Methods section.*
☒	☐	A description of all covariates tested
☒	☐	A description of any assumptions or corrections, such as tests of normality and adjustment for multiple comparisons
☐	☒	A full description of the statistical parameters including central tendency (e.g. means) or other basic estimates (e.g. regression coefficient) AND variation (e.g. standard deviation) or associated estimates of uncertainty (e.g. confidence intervals)
☐	☒	For null hypothesis testing, the test statistic (e.g. *F*, *t*, *r*) with confidence intervals, effect sizes, degrees of freedom and *P* value noted<br>*Give P values as exact values whenever suitable.*
☒	☐	For Bayesian analysis, information on the choice of priors and Markov chain Monte Carlo settings
☒	☐	For hierarchical and complex designs, identification of the appropriate level for tests and full reporting of outcomes
☒	☐	Estimates of effect sizes (e.g. Cohen's *d*, Pearson's *r*), indicating how they were calculated

Our web collection on statistics for biologists contains articles on many of the points above.

Software and code

Policy information about availability of computer code

Data collection	MetaMorph (v7.10.2.240) (Molecular Devices LLC) was used to collect microscopy data.<br>Biotek Gen5 (v3.02) was used to collect growth curve data.
Data analysis	ImageJ (v1.53) and MicrobeJ (v5.13l) were used for image analyses.<br>PEAR (v0.9.11) and USEARCH (v11.0.667) were used for Illumina read clustering.<br>FlowJo (v10.0.7) was used for cytometry date analyses.<br>Geneious Primer (v2022.2.2) was used for RNA-seq analysis.

For manuscripts utilizing custom algorithms or software that are central to the research but not yet described in published literature, software must be made available to editors and reviewers. We strongly encourage code deposition in a community repository (e.g. GitHub). See the Nature Portfolio guidelines for submitting code & software for further information.

Data

Policy information about availability of data

All manuscripts must include a data availability statement. This statement should provide the following information, where applicable:

- Accession codes, unique identifiers, or web links for publicly available datasets
- A description of any restrictions on data availability
- For clinical datasets or third party data, please ensure that the statement adheres to our policy

High-throughput data generated in this study is available with NCBI BioSample accessions SAMN32730695 and SAMN32730696.

Human research participants

Policy information about studies involving human research participants and Sex and Gender in Research.

Reporting on sex and gender	N/A
Population characteristics	N/A
Recruitment	N/A
Ethics oversight	N/A

Note that full information on the approval of the study protocol must also be provided in the manuscript.

Field-specific reporting

Please select the one below that is the best fit for your research. If you are not sure, read the appropriate sections before making your selection.

☒ Life sciences ☐ Behavioural & social sciences ☐ Ecological, evolutionary & environmental sciences

For a reference copy of the document with all sections, see nature.com/documents/nr-reporting-summary-flat.pdf

Life sciences study design

All studies must disclose on these points even when the disclosure is negative.

Sample size	Sample sizes were chosen based on the number needed to reliably determine differences between groups. All experiments were performed 2-4 times independently.
Data exclusions	No data exclusion was performed.
Replication	All experimental findings were repeated at least twice. Exact biological repeats are indicated. All reported results were successfully reproduced.
Randomization	No experimental groups or control groups were subjectively chosen and there are no covariates to control for as experiments were done in isogenic strains. No experiments required randomization.
Blinding	Blinding was not relevant because all data were obtained objectively and had strong effect sizes.

Reporting for specific materials, systems and methods

We require information from authors about some types of materials, experimental systems and methods used in many studies. Here, indicate whether each material, system or method listed is relevant to your study. If you are not sure if a list item applies to your research, read the appropriate section before selecting a response.

Materials & experimental systems

n/a	Involved in the study
☐	☒ Antibodies
☒	☐ Eukaryotic cell lines
☒	☐ Palaeontology and archaeology
☒	☐ Animals and other organisms
☒	☐ Clinical data
☒	☐ Dual use research of concern

Methods

n/a	Involved in the study
☒	☐ ChIP-seq
☐	☒ Flow cytometry
☒	☐ MRI-based neuroimaging

Antibodies

Antibodies used

6x-His Tag Monoclonal Antibody (clone HIS.H8, Invitrogen Cat#: MA1-21315).
Anti-RpoA antibody (Biolegend Cat#: 663104).
Anti-FLAG antibody (Sigma Cat#: F1804).
Anti-DnaK antibody (Abcam Cat#: ab69617).
Anti-DnaJ antibody (enzo life sciences Cat#:ADI-SPA-410-F).
Goat anti-Mouse IgG Secondary Antibody, HRP (Invitrogen Cat#: 32430).
Goat anti-Rabbit IgG Secondary Antibody, HRP (Invitrogen Cat#: 32460).

Validation

All antibodies used in this study are commercial, standard antibodies routinely used in bacterial studies. Manufacturers specify that antibody reactivity is determined by testing in at least one approved application (e.g., western blot). Antibodies were used according to the manufacturer's guidelines. We performed internal validations of antibodies against negative control strains.

Flow Cytometry

Plots

Confirm that:

☒ The axis labels state the marker and fluorochrome used (e.g. CD4-FITC).

☒ The axis scales are clearly visible. Include numbers along axes only for bottom left plot of group (a 'group' is an analysis of identical markers).

☒ All plots are contour plots with outliers or pseudocolor plots.

☒ A numerical value for number of cells or percentage (with statistics) is provided.

Methodology

Sample preparation

Strain ML-4048 or ML-4050 with plasmids ML-4052 to ML-4055 or ML-4058 were grown overnight at 37 oC in LB supplemented with appropriate antibiotics. Cultures were diluted 1:500 in medium supplemented with 100 ng/µL aTc to induce expression of the random genes (or an empty vector) and grown for 30 minutes at 37 oC. Then, either 0.2% arabinose or 100 µM vanillate was added to induce the expression of msfGFP. Cultures were grown an additional 4.5 hours at 37 oC, then diluted 1:40 into PBS supplemented with a high concentration of (0.5 g/L) kanamycin to stop translation, and incubated at room temperature for 10 min. Then, fluorescence was measured on a Miltenyi MACSQuant VYB.

Instrument

Miltenyi MACSQuant VYB (Miltenyi Biotec)

Software

FlowJo

Cell population abundance

30,000 cells were measured per replicate and at least 18,000 were left post-gating.

Gating strategy

Initial gating was performed using SSC-A and FSC-A and then single bacterial cells were identified using parameters SSC-A and SSC-H.

☒ Tick this box to confirm that a figure exemplifying the gating strategy is provided in the Supplementary Information.
