# Supplementary figures and images for: Selection of a de novo gene that can promote survival of Escherichia coli by modulating protein homeostasis pathways
Source: Nat Ecol Evol. 2023 Nov 9;7(12):2067–79. doi: 10.1038/s41559-023-02224-4 (PMC10697842; doi:10.1038/s41559-023-02224-4)

## Source Data Figure 2

2D

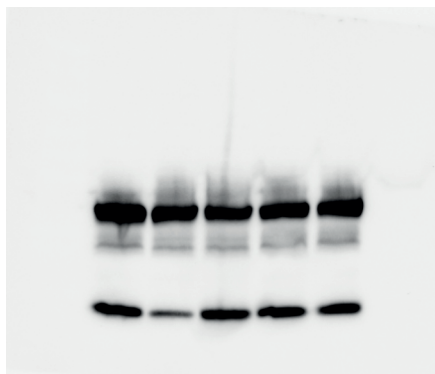

2F 1

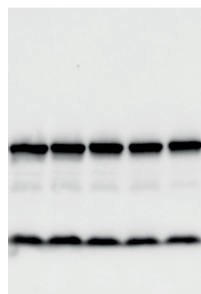

2F 2

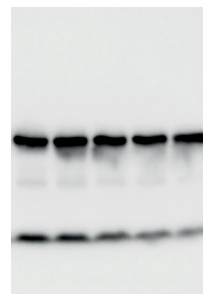

2G 1

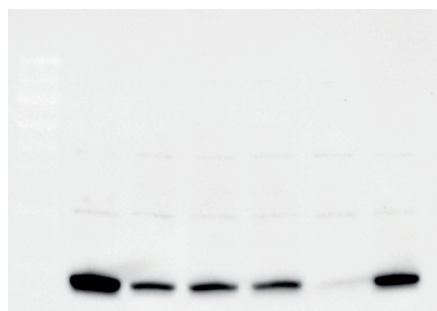

2G 2

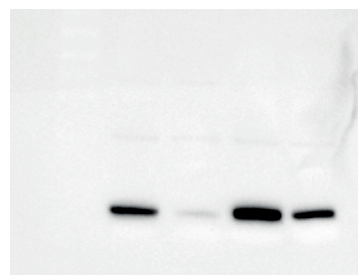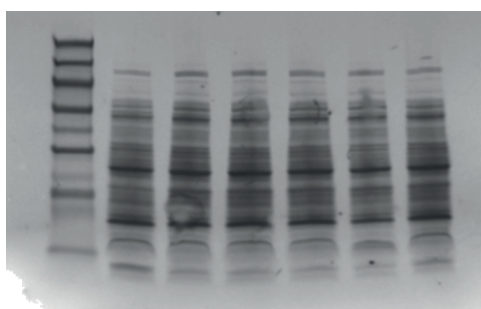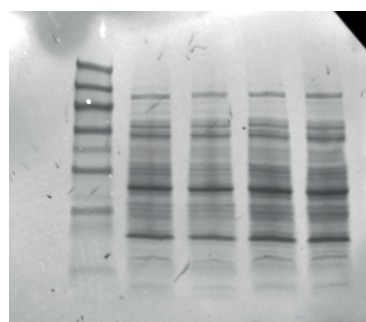

Supplement: Supplementary file 4 — Unprocessed western blots. [file 41559_2023_2224_MOESM4_ESM.pdf]

## Source Data Figure 4

4G

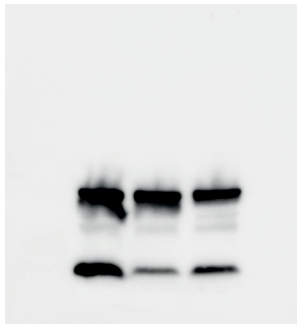

Supplement: Supplementary file 5 — Unprocessed western blots. [file 41559_2023_2224_MOESM5_ESM.pdf]

## Source Data Figure 5

5B

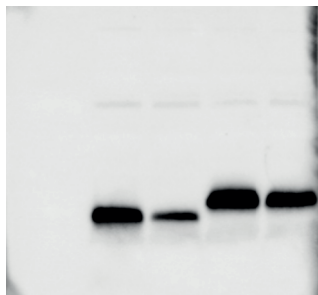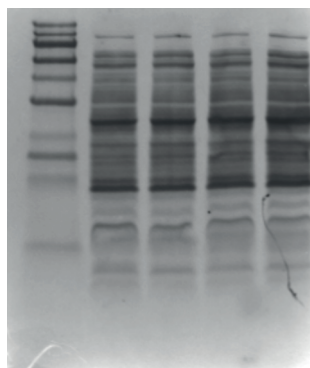

Supplement: Supplementary file 6 — Unprocessed western blots. [file 41559_2023_2224_MOESM6_ESM.pdf]

## Source Data Figure 6

6F

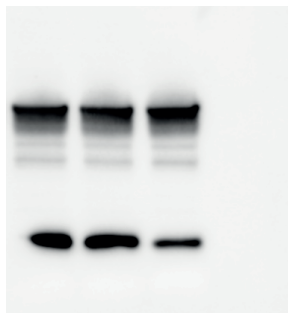

Supplement: Supplementary file 7 — Unprocessed western blots. [file 41559_2023_2224_MOESM7_ESM.pdf]

## Source Data Extended Data Figure 4

4A

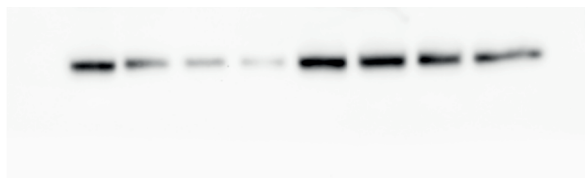

4B

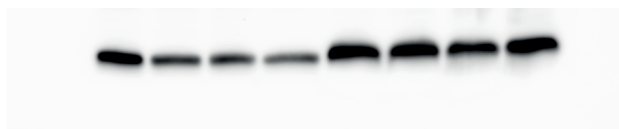

Supplement: Supplementary file 8 — Unprocessed western blots. [file 41559_2023_2224_MOESM8_ESM.pdf]

## Source Data Extended Data Figure 5

5E

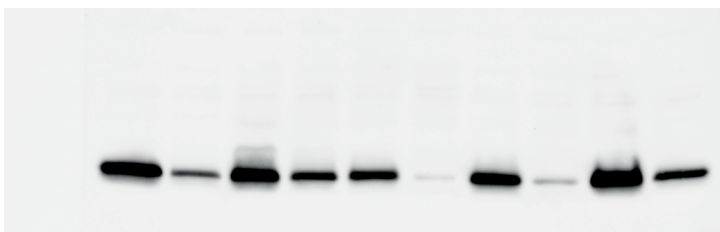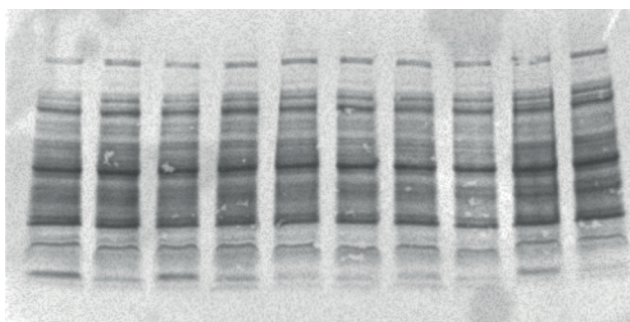

5F

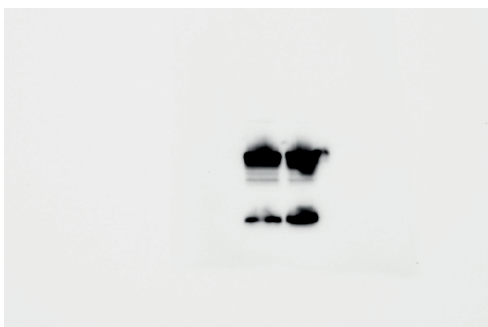

Supplement: Supplementary file 9 — Unprocessed western blots. [file 41559_2023_2224_MOESM9_ESM.pdf]

## Source Data Extended Data Figure 8

8B

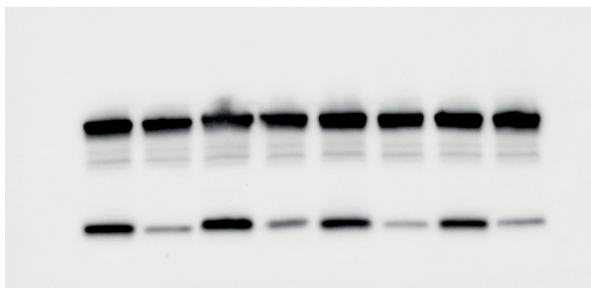

Supplement: Supplementary file 10 — Unprocessed western blots. [file 41559_2023_2224_MOESM10_ESM.pdf]

## Source Data Extended Data Figure 9

9A

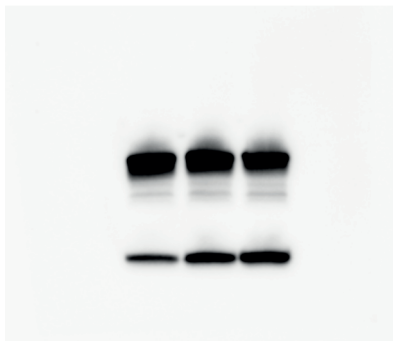

9D 1

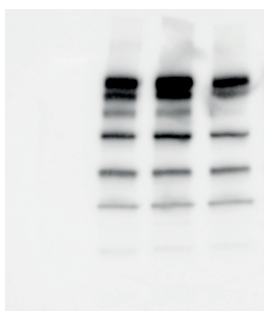

9D 2

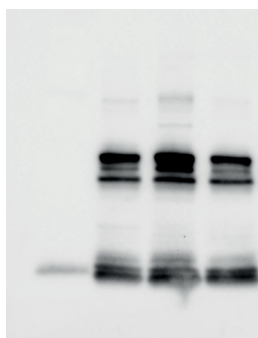

9D 3

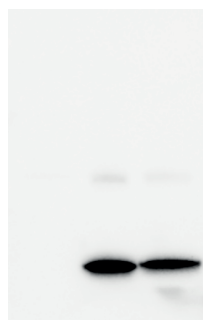

Supplement: Supplementary file 11 — Unprocessed western blots. [file 41559_2023_2224_MOESM11_ESM.pdf]
